# Proteins recruited by SH3 domains of Ruk/CIN85 adaptor identified by LC-MS/MS

**DOI:** 10.1186/1477-5956-7-21

**Published:** 2009-06-16

**Authors:** Serhiy Havrylov, Yuriy Rzhepetskyy, Agata Malinowska, Lyudmyla Drobot, Maria Jolanta Redowicz

**Affiliations:** 1 Laboratory of Molecular Basis of Cell Motility, Nencki Institute of Experimental Biology, 3 Pasteur str., 02-093 Warsaw, Poland; 2 Laboratory of Mass Spectrometry, Institute of Biochemistry and Biophysics, 5a Pawinskiego str., 02-106 Warsaw, Poland; 3 Palladin Institute of Biochemistry, 9 Leontovich str., Kyiv 01601, Ukraine

## Abstract

**Background:**

Ruk/CIN85 is a mammalian adaptor molecule with three SH3 domains. Using its SH3 domains Ruk/CIN85 can cluster multiple proteins and protein complexes, and, consequently, facilitates organisation of elaborate protein interaction networks with diverse regulatory roles. Previous research linked Ruk/CIN85 with the regulation of vesicle-mediated transport and cancer cell invasiveness. Despite the recent findings, precise molecular functions of Ruk/CIN85 in these processes remain largely elusive and further research is hampered by a lack of complete lists of its partner proteins.

**Results:**

In the present study we employed a LC-MS/MS-based experimental pipeline to identify a considerable number (over 100) of proteins recruited by the SH3 domains of Ruk/CIN85 *in vitro*. Most of these identifications are novel Ruk/CIN85 interaction candidates. The identified proteins have diverse molecular architectures and can interact with other proteins, as well as with lipids and nucleic acids. Some of the identified proteins possess enzymatic activities. Functional profiling analyses and literature mining demonstrate that many of the proteins recruited by the SH3 domains of Ruk/CIN85 identified in this work were involved in the regulation of membranes and cytoskeletal structures necessary for vesicle-mediated transport and cancer cell invasiveness. Several groups of the proteins were also associated with few other cellular processes not previously related to Ruk/CIN85, most prominently with cell division.

**Conclusion:**

Obtained data support the notion that Ruk/CIN85 regulates vesicle-mediated transport and cancer cell invasiveness through the assembly of multimeric protein complexes governing coordinated remodelling of membranes and underlying cytoskeletal structures, and imply its important roles in formation of coated vesicles and biogenesis of invadopodia. In addition, this study points to potential involvement of Ruk/CIN85 in other cellular processes, chiefly in cell division.

## Background

Ruk/CIN85 is a mammalian adaptor molecule with three SH3 domains. In addition to the SH3 domains located at the N-terminal part of the protein, Ruk/CIN85 contains other protein interaction modules, namely proline-rich sequences and a coiled-coil region (see Additional file 1). By sequence homology and domain architecture Ruk/CIN85 is closely related to another mammalian adaptor molecule CD2AP/CMS [1]. Due to the similarity of their SH3 domains, Ruk/CIN85 and CD2AP/CMS recognise the same atypical proline-arginine PX(P/A)XXR motif and reveal partially overlapping specificity towards interaction partners [2,3]. Presence of the multiple protein interaction modules, particularly of the SH3 domains, endows both these adaptors with the capacity to engage a variety of proteins with wide range of activities and assemble the multimeric protein complexes of various composition and cellular functions. In other words, via the clustering of multiple proteins and protein complexes, Ruk/CIN85 and CD2AP/CMS facilitate the organisation of elaborate protein interaction networks with diverse regulatory roles.

Previous research linked Ruk/CIN85 with various cellular functions such as signal transduction, vesicle-mediated transport and membrane trafficking, rearrangement of cytoskeletal structures, regulation of cell adhesion, cancer cell invasiveness and cell death [1,4-9]. Recent studies indicate that contribution of Ruk/CIN85 to regulation of membrane trafficking and cancer cell invasiveness is particularly important [9-11]. Localisation studies show that Ruk/CIN85 acts at different cellular membranes and membrane trafficking compartments. In our previous study we have observed endogenous Ruk/CIN85 primarily at the COPI-coated membranes and the vesicles of the Golgi complex, and occasionally at the edges of protrusive actin-rich structures of migrating cells [11]. Ruk/CIN85 also functions as an important component of invadopodia – adhesive actin-rich membrane protrusions of invasive cancer cells [9].

Despite significant attention paid by researchers to Ruk/CIN85 in recent years, the precise molecular functions of this adaptor molecule remain largely elusive and, in certain cases, discrepant. For example, the participation of Ruk/CIN85 in endocytic membrane transport has been challenged by several recent studies [12,13]. Additional experiments are necessary to resolve this controversy. Further research on the molecular functions of Ruk/CIN85 is hampered not only by insufficient *in vivo *functional studies but, to a great extent, also by a lack of complete list of proteins recruited by this adaptor. Since activity of Ruk/CIN85 in different cellular processes is determined solely by its ability to cluster other protein molecules, identification of the complete list of proteins recruited by each of the protein interaction modules found in Ruk/CIN85 may provide a wealth of information for the better understanding of its molecular functions.

In the present study we identified lists of proteins recruited *in vitro *by all of the three SH3 domains of Ruk/CIN85 using the high-throughput technique combining affinity purification of interaction partners from cell lysates with following liquid chromatography-coupled tandem mass spectrometry (LC-MS/MS). Subsequently, we subjected the obtained lists to functional profiling and association network analysis to infer potential functions of protein interaction networks organised on the SH3 domains of this adaptor molecule.

## Results

### Variety and modular architectures of the proteins recruited by the SH3 domains of Ruk/CIN85

In the present study we utilised the liquid chromatography-coupled tandem mass spectrometry (LC-MS/MS)-based approach to identify the lists of the proteins recruited by the SH3 domains of Ruk/CIN85 *in vitro*. This sensitive and precise tool for detection of multiple proteins in complex mixtures has not been used to study interactions mediated by Ruk/CIN85 so far.

Using the experimental pipeline comprised of GST pull-downs, SDS-PAGE and LC-MS/MS, we identified 107 proteins recruited by the SH3 domains of Ruk/CIN85 (Table 1, see Additional file 2 for more details). Most of these identifications (approximately 90%) were novel interaction candidates, which had not been previously reported to bind Ruk/CIN85, neither in direct nor in indirect ways. Only twelve of the identified proteins including GTPase-activating proteins RICH1 and ASAP1/AMAP1, adaptor/scaffold molecules Sb1, CFBP, AIP1, Dab2, 3BP-2 and Atx2, E3 ubiquitin ligases c-Cbl, Cbl-b, and two inositol-5'-phosphatases synaptojanins 1 and 2 were previously implicated in the interactions with Ruk/CIN85 (see ref. [14] for most recent review).

**Table 1:** Proteins recruited by the SH3 domains of Ruk/CIN85 identified by LC-MS/MS and classified according to modular architectures.

*GI*	*Name*	*Score*	*Mr*	*u.p.*	*% c.*	*A*	*B*	*C*	*c.s.*	*k/n*	*Modules/domains*
**1. Proteins with lipid interaction modules**											
											
*1.a. with lipid and protein interaction modules*											
gi|74132096	Tks4	720	101847	21	27	•	•	•	•		PX, SH3, SH3, PR, SH3
gi|38424073	LL5α	445	152038	8	6	•	•	•	•		FHA, CC, CC, CC, CC, CC, CC, PH
gi|19923493	PEPP-2	326	128012	8	9			•			WW, WW, PH, CC, CC
gi|31657094	Anillin	280	125490	9	9	•	•	•	•		CC, PH
gi|3023207	3BP-2	160	62660	3	10	•			•	•	PH, PR, SH2
gi|82581557	Lamellipodin	142	141762	3	3	•			•		CC, RA, PH, PR, Paxillin, Paxillin, Paxillin
gi|84029396	LL5β	128	142812	5	4	•	•	•	•		CC, CC, CC, CC, CC, PH
*1.b. with lipid interaction modules and GAP activity*											
gi|54860079	RICH1	1153	95776	26	44	•	•	•	•	•	BAR, RhoGAP, PR
gi|92091600	ARAP1	481	163743	13	10	•	•	•	•		SAM, PR, PH, PH, ArfGAP, PH, PH, RhoGAP, RA, PH
gi|119370361	ASAP1	202	126363	6	6	•	•	•	•	•	BAR, PH, ArfGAP, Ank, PR, SH3
gi|21361397	MgcRacGAP	157	71666	3	6			•			RGS, CC, C1, RhoGAP
gi|42475970	SYDE1	127	80428	2	3			•	•		PR, CaLB, RhoGAP
gi|4758808	RASAL2	64	129618	2	2			•	•		PH, CaLB, RhoGAP/RasGAP, CC
*1.c. with lipid interaction modules and GTPase activity*											
gi|56549121	Dynamin 2	1002	98345	13	24	•		•	•		Dynamin GTPase, Dynamin central, PH, GED, PR
gi|59853099	Dynamin 1	559	97746	9	18	•		•	•		Dynamin GTPase, Dynamin central, PH, GED, PR
											
**2. Proteins with protein interaction modules**											
*2.a. with actin-binding modules*											
gi|31324577	MIRab13	494	94352	10	13	•	•	•	•		CH, Znf-LIM, PR, CC, CC
gi|51702526	N-WASP	411	55192	14	31	•		•	•		EVH1, PBD/CRIB, PR, WH2, WH2
gi|19923777	GAR22	366	73328	5	10	•			•		CH, GAS2, PR
gi|60416429	WIP	295	51300	8	20	•		•	•		WH2, PR
gi|55741671	LIMCH1	271	122788	6	7	•	•		•		CH, CC, CC, CC, Znf-LIM
gi|18959210	WIRE	196	46317	5	14	•		•	•		WH2, PR
gi|48428650	Synaptopodin	173	99915	4	6	•		•	•		Synaptopodin, PR
*2.b. with MT-binding modules*											
gi|20143967	MKLP-1	304	111016	6	8	•	•	•			Kinesin, CC
gi|20455500	MAP4	284	121457	10	12	•		•			Tau
gi|4505101	E-MAP-115	152	84116	3	5		•			•	CC, PR, E-MAP-115
gi|4506039	PRC1	128	72304	4	7	•					MAP65/ASE1
gi|39963533	MAP7D3	102	90256	2	2		•			•	CC, CC, CC, E-MAP-115
*2.c. with modules involved in MT nucleation*											
gi|5453660	GCP3	513	104304	10	16	•			•	•	Spc97/Spc98, CC
gi|5729840	GCP2	275	103096	5	6	•			•	•	CC, Spc97/Spc98
gi|38454194	GCP4	71	76483	3	4				•		Spc97/Spc98
*2.d. with GTP/protein interaction modules*											
gi|6683817	Septin 9	1357	65614	22	43	•	•	•	•		Septin
gi|4758158	Septin 2	711	41679	9	32	•		•			Septin, CC
gi|8922712	Septin 11	553	49652	8	32	•	•	•			Septin, CC
gi|57209157	Septin 6	388	49614	3	20	•	•	•			Septin, CC, CC
gi|21945064	Septin 10	328	53016	8	25	•	•	•			Septin, CC
gi|67472677	Septin 7	202	50933	6	12	•	•	•	•		Septin, CC, CC
gi|45645200	Septin 8	173	56234	3	16	•	•	•			Septin, CC, CC
*2.e. other with protein interaction modules*											
gi|19920317	CKAP4	846	66097	17	35		•	•	•		PR, TM, Spectrin, CC, CC, CC, STAT, CC
gi|12644198	CDC27	500	92893	10	18		•	•	•		TPR, TPR, TPR, TPR, TPR, TPR, TPR, CC, TPR
gi|13375926	Vps37B	299	31345	4	22	•	•	•	•		Modr, PR
gi|38045892	ELKS	296	113964	8	9	•		•			CC, CC, CC, CC, CC, Prf, Prf, CC, CC, CC, CC, CC
gi|116241251	APC7	278	63720	4	10		•	•			TPR, TPR, TPR, TPR
gi|46255026	KCTD3	272	89613	7	11	•	•	•			BTB/POZ, WD40, WD40, WD40
gi|37537859	APC8	239	69040	4	10		•	•			TPR, TPR, TPR, TPR, TPR, TPR
gi|119577384	Sb1	234	58997	5	11	•	•	•	•	•	WD40, WD40, WD40
gi|62751805	SH3D19	222	86713	5	7	•	•	•	•		PR, SH3, SH3, SH3, SH3, SH3
gi|37537862	APC4	215	92789	5	7		•	•			WD40
gi|63054866	CARMIL	212	148193	4	3	•	•		•		LRR, LRR, CC
gi|8923243	Palmdelphin	184	62777	3	7	•	•	•	•		CC, CC, CC, Paralemmin
gi|26454712	SLAIN2	172	62773	3	9	•		•			CC
gi|24308440	CFBP	165	29107	2	10	•			•	•	PR
gi|112734870	LOC57648	161	113392	4	6	•		•	•		PR
gi|6424942	AIP1	154	96646	2	3	•	•		•	•	BRO1, CC, CC, PR
gi|20127553	APC5	147	85707	3	5			•			TPR, TPR
gi|31044432	hLEM2	136	57339	3	7		•		•		LEM, TM, TM
gi|4758116	Dystroglycan 1	134	97862	2	3			•	•		Cadherin, Ig-like, Cadherin, PR, TM, PR
gi|38173707	BAT2	132	229181	3	2	•		•			BAT2, PR, CC, PR
gi|83779014	Katanin p80	128	73257	2	5	•		•	•		WD40, WD40, WD40, WD40, WD40, WD40, PR
gi|14249168	DDA3	127	35725	3	12	•	•	•	•		CC, PR
gi|13994353	KCTD10	120	35809	2	8		•		•		BTB/POZ
gi|4503251	Dab2	113	82511	3	4			•	•	•	PTB/PID, PR, PfkB
gi|116242719	POM121	113	125382	2	3	•		•	•		PR
gi|6005830	Plakophilin 3	111	87485	2	4	•			•		Arm, Arm, Arm
gi|115392150	FAM83G	109	91064	3	3		•		•		DUF1669, PR
gi|10334526	WTAP	107	43557	2	6			•			CC, CC, CC, CC
gi|5533375	APC6	96	66575	2	4		•				TPR, TPR, TPR
gi|44917604	srGAP1	94	125099	2	1	•		•	•		CC, FCH, CC, RhoGAP, SH3, CC
gi|52000732	Atx2	88	140681	2	2	•			•	•	PR, CC, Like-Sm, Atx2, PR, CC, PAM2, PR
gi|24850113	SHCBP1	72	76669	2	3			•			Pbh1, Pbh1, Pbh1, Pbh1, Pbh1, CC
											
**3. Proteins with nucleic acid interaction modules**											
*3.a. with RNA-binding modules*											
gi|118572613	SRm300	602	300179	11	5	•		•	•		Cwf21, PR, atypical RNA-binding motif
gi|5730027	Sam68	431	48311	8	21	•	•	•	•		PR, KH, PR
gi|14165437	hnRNPK	236	51281	5	17			•	•		ROK, KH, KH, PR, KH
gi|4504715	PABPC4	136	71080	3	4	•		•	•		RRM, RRM, RRM, CC, RRM, PABP/HYD
gi|119612222	PABPC1	136	47647	3	10	•		•			RRM, RRM, RRM, RRM, PABP/HYD
gi|13937888	hnRNPC	128	33635	3	12	•			•		RRM, CC, CC
gi|72534660	9G8	118	27578	2	8	•					RRM, Znf-CCHC
*3.b. with RNA/DNA-binding modules*											
gi|46852388	CARP1	655	133423	8	9	•		•	•		CSP, CC, SAP, CC, CC, CC
gi|42542379	SRm160	209	102331	6	9	•		•	•		PWI, CC, PR, PR, CC
gi|23510448	MCM5	150	83301	4	7		•				OB-fold, MCM
gi|115430211	PHACTR4	127	78391	2	4	•		•	•		RPEL, PR, RPEL, Sigma3/Sigma4, RPEL
gi|4506583	RP-A	102	68723	2	3		•		•		OB-fold, OB-fold, OB-fold, OB-fold
*3.c. with RNA/protein-binding modules*											
gi|24638431	Caskin 2	357	127204	10	11	•	•	•	•		ANK, ANK, ANK, ANK, ANK, ANK, SH3, SAM, SAM, PR
gi|39930517	Atherin	79	56189	2	5			•	•		PR, SAM
gi|29294627	Liprin β1	61	113680	2	2		•				CC, CC, Intergase, CC, SAM, SAM, SAM
*3.d. with DNA-binding modules*											
gi|4050036	LEDGF	488	60093	8	19		•	•	•		PWWP, AT-hook, AT-hook, AT-hook, CC, TFS2N
gi|33872239	C14orf43	138	112905	3	3		•	•	•		PR, ELM2, SANT
gi|37693995	KLF13	77	31617	2	8			•	•		PR, Znf-C2H2, Znf-C2H2, Znf-C2H2
gi|42544179	CIZ1	74	101066	2	3			•	•		CC, Znf-C2H2, Znf-C2H2, CC, Znf-C2H2
											
**4. Proteins with enzymatic activities**											
*4.a. with inositol 5'-phosphatase activity*											
gi|4755142	SHIP2	726	139297	17	17	•		•	•		SH2, IPP, PR, SAM
gi|26190608	Synaptojanin 2	697	166575	22	17	•	•	•	•	•	Synaptojanin, IPP, RRM, PR
gi|8134730	Synaptojanin 1	100	174378	3	2			•	•	•	Synaptojanin, IPP, RRM, PR
*4.b. with protein kinase activity*											
gi|62362414	c-Abl	241	123595	6	9	•	•	•	•		SH3, SH2, Tyr kinase, F-actin binding
gi|68303575	CKIa	237	42195	4	14		•				Ser/Tre kinase
gi|4506735	S6K a4	144	86122	3	5	•			•		Ser/Tre kinase, Ser/Tre kinase
gi|5453860	PCTAIRE-1	116	55909	3	8		•	•			Ser/Tre kinase
*4.c. with E3 ubiquitin ligase activity*											
gi|52426745	c-Cbl	353	100881	9	15	•		•	•	•	Cbl N, EF, SH2, Znf-RING, PR, UBA
gi|116734704	IRF2-BP2	311	61728	5	16	•		•	•		Znf-RING
gi|862407	Cbl-b	298	110893	8	11	•	•	•	•	•	Cbl N, EF, SH2, Znf-RING, PR, UBA
gi|24308115	IRF2-BP1	211	62561	3	5	•			•		Znf-RING
*4.d. with other ensymatic activities*											
gi|4503351	MCMT	286	185388	9	6			•	•		DMAP1, CC, Znf-CXXC, BAH, BAH, DNMT
gi|47678395	DDX17	269	73138	6	12	•		•	•		Q motif, helicase N, helicase C, PR
gi|5901990	Katanin p60	148	56214	3	7	•	•	•	•		AAA ATPase, Vps4
gi|4757810	ATP synthase a	108	59828	3	6		•	•			F1 ATPase N, F1 AAA ATPase, F1 ATPase C
gi|42476028	ATAD3A	100	71598	3	4		•				CC, CC, CC, CC, AAA ATPase
gi|8922976	DDX28	88	59743	2	4		•		•		Q motif, helicase N, helicase C
											
**5. Other proteins**											
gi|20127556	GTSE-1	176	77081	4	8	•	•		•		none
gi|47124471	Colligin	113	46525	2	6		•				Serpin, ER-targeting

*GI *– protein genbank identifier; *Name *– short protein name; *Score *– Mascot protein score; *Mr *– protein molecular weight; *u.p*. – number of unique peptides matched to protein sequence; *% c*. – % of protein sequence covered by matched peptides. Dots in columns *A*, *B*, or *C *indicate that protein was recruited by SH3A, SH3B and/or SH3C domains respectively. Dot in column *c.s*. indicates presence of potential recognition consensus site for the SH3 domains of Ruk/CIN85 in identified protein. Dot in column *k/n *indicates that identified protein was previously known to interact with Ruk/CIN85 adaptor. The short names of modules found in identified proteins are listed in column *Modules/domains*. Long names of modules/domain abbreviations are listed in the Additional file 3.

**Figure 1:**
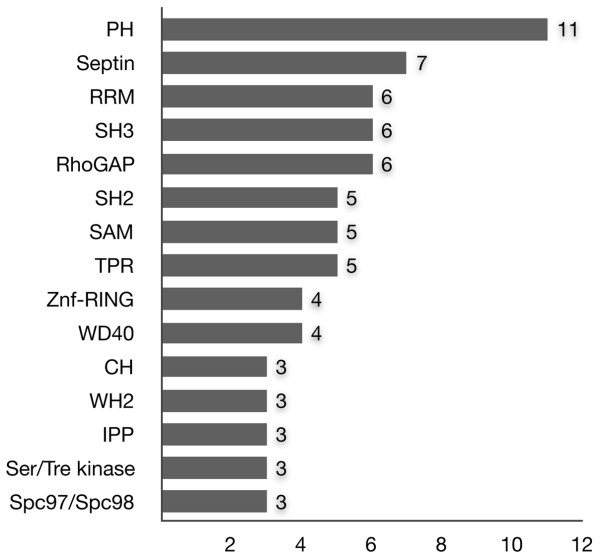
**Occurrence of certain modules in the identified proteins recruited by the SH3 domains of Ruk/CIN85**. PH – pleckstrin homology domain; Septin – cell division/GTP-binding septin family region; RRM – RNA recognition motif; SH3 – Src homology 3 domain; RhoGAP – Rho GTPase activating domain; SH2 – Src homology 2 domain; SAM – sterile alpha motif; TPR – tetratricopeptide repeat region; Znf-RING – zinc finger RING-type domain; WD40 – WD40/WD/beta-transducin repeat; CH – calponin-homology actin-binding domain; WH2 – WASP homology domain 2; IPP – inositol polyphosphate phosphatase domain; Ser/Tre kinase – serine/threonine protein kinase domain; Spc97/Spc98 – Spc97/Spc98 family region.

For initial classification, the identified proteins were grouped according to their modular architectures. Resulting classification distinguished the four major protein classes encompassing (1) proteins containing lipid interaction modules, (2) proteins with modules able to bind nucleic acids, (3) proteins containing only protein interaction modules and (4) proteins with enzymatic activities (Table 1). Within this classification, group 3 was the largest one. Except for ubiquitous coiled-coil and proline-rich regions, these proteins frequently had SH3, SH2, TPR and WD40 domains known to be involved in protein-protein interactions (Fig. 1). This group also harboured the distinct subgroups of proteins with actin binding modules (mostly CH and WH2 domains), modules required for interaction with microtubules or for microtubule assembly, and also the subgroup of atypical cytoskeletal GTP-binding proteins called septins.

More than a half of proteins from the lipid-binding group were classified as adaptor molecules with lipid and protein interaction modules. Except for two dynamin GTPases, the rest of proteins (approximately 80%) with lipid interaction modules were represented by the GTPase-activating proteins (GAPs) for Rho family GTPases. Most of the lipid-binding candidates contained PH domains – lipid/protein interaction modules specific towards phosphoinositides, and frequently also towards proteins. Notably, in comparison with the other modules (excluding coiled-coil and proline-rich regions), PHs were the most common domains in the identified proteins (Fig. 1).

The proteins with enzymatic activities were classified as inositol-5'-phosphatases, protein kinases and E3 ubiquitin ligases. Several other proteins of this group contained core ATPase domains, and two belonged to RNA-dependent helicases (see Table 1).

Surprisingly, the significant group of the identified proteins contained modules implicated in interactions with nucleic acids. This group included proteins with both RNA and DNA interaction modules, with prevalence of RNA-binding molecules. RRM RNA-binding domains and SAM dual specificity RNA/protein-binding domains were among interaction modules frequently found in the identified proteins (Fig. 1). Notably, RRMs were found not only in the novel Ruk/CIN85 interaction candidates, but also in synaptojanins 1 and 2 – the two known interaction partners of this adaptor molecule (see Table 1).

### Potential recognition consensus sites for the SH3 domains of Ruk/CIN85 in the identified proteins

To define which of the identified proteins were likely to interact with Ruk/CIN85 directly we analysed their amino acid sequences for the presence of potential recognition consensus sites for the SH3 domains of Ruk/CIN85 using Scansite software. For this purpose we employed the experimental data on consensus peptide composition for the SH3 domains of Ruk/CIN85 reported previously by Kurakin et al. [2] to design matrices used for analysis (see Experimental Procedures section for details). Scansite analysis performed with these matrices identified potential recognition consensus sites for the SH3 domains of Ruk/CIN85 in 77 of 107 proteins (Table 1). Provided that the samples obtained by the affinity purification on the SH3 domains of Ruk/CIN85 were expected to be significantly enriched in proteins containing binding sites for these domains, results of the analysis were not surprising, and indicated that up to 71% of proteins found in the samples might bind to the SH3 domains of Ruk/CIN85 directly. Other 29% of the identifications might combine proteins co-purified with the direct interaction partners, and in some cases, minor impurities.

### Overlapping specificity of the SH3 domains of Ruk/CIN85 and evolutionary origin of Ruk/CIN85

Among the proteins with potential recognition consensus sites for the SH3 domains of Ruk/CIN85, and hence likely to interact with these domains directly, more then a half (64%) was identified simultaneously in the samples for two of three different SH3 domains of Ruk/CIN85, and almost one third (27%) was found in the samples for all the three domains (Table 1). These data indicate that the SH3 domains of Ruk/CIN85 are redundant in terms of their affinity towards interaction partners, which reflects similarity of recognition consensus sequences shared by these domains [2]. Interestingly, although all of the SH3 domains of Ruk/CIN85 recognise the same peptide ligand core sequence, PX(P/A)XXR, SH3A and SH3C domains share significantly more interaction partners, than SH3A or SH3C with SH3B do (Table 1). Of even more interest, recent study utilising NMR spectroscopy have demonstrated that despite sharing the same peptide ligand core sequence, SH3A and SH3B domains of Ruk/CIN85 use different mechanisms to bind their ligands [15].

**Figure 2:**
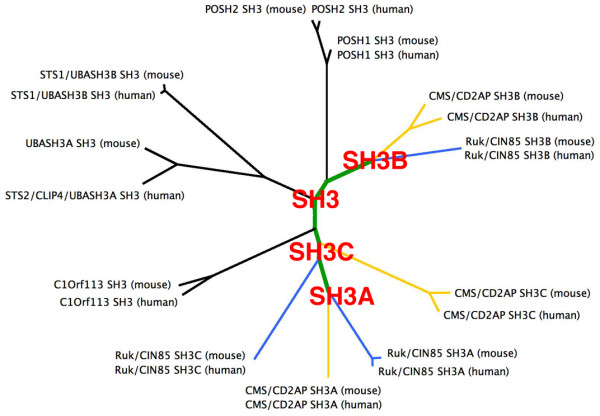
**Phylogenetic analysis of the SH3 domains from murine and human proteins**. Radial phylogram of the clade harbouring the SH3 domains of Ruk/CIN85 which demonstrates common evolution of the SH3 domains of Ruk/CIN85 and CD2AP/CMS in early mammalian ancestors. For the phylogenetic tree of all of the SH3 domains found in mice and men used in the analysis refer to the Additional file 4.

One possible explanation for the higher similarity of SH3A and SH3C domains with respect to their interaction affinities might stem from their evolutionary origin. To verify this assumption, we performed the phylogenetic analysis of the sequences of all mouse and human SH3 domains deposited in Pfam database [16]. The analysis revealed that all of the SH3 domains of Ruk/CIN85 were closely related, and, probably, had originated during the early evolution of mammals via the sequential duplication of a progenitor SH3 domain to generate initially ancestor SH3B and SH3C domains, and then ancestor SH3A domain via duplication of the SH3C ancestor (Fig. 2; see Additional file 4 for the whole phylogenetic tree of all SH3 domains analysed). Finally, the progenitor adaptor protein containing three SH3 domains present in early mammals gave origin to Ruk/CIN85 and CD2AP/CMS, the two closely homologous adaptor proteins with similar domain structure found in contemporary mammal species. According to this scenario, SH3A and SH3C domains are more evolutionary related, and therefore, likely to have more similar structure and, hence, to be more redundant in their interaction affinities.

### Functional clusters of the identified interaction candidates

**Figure 3:**
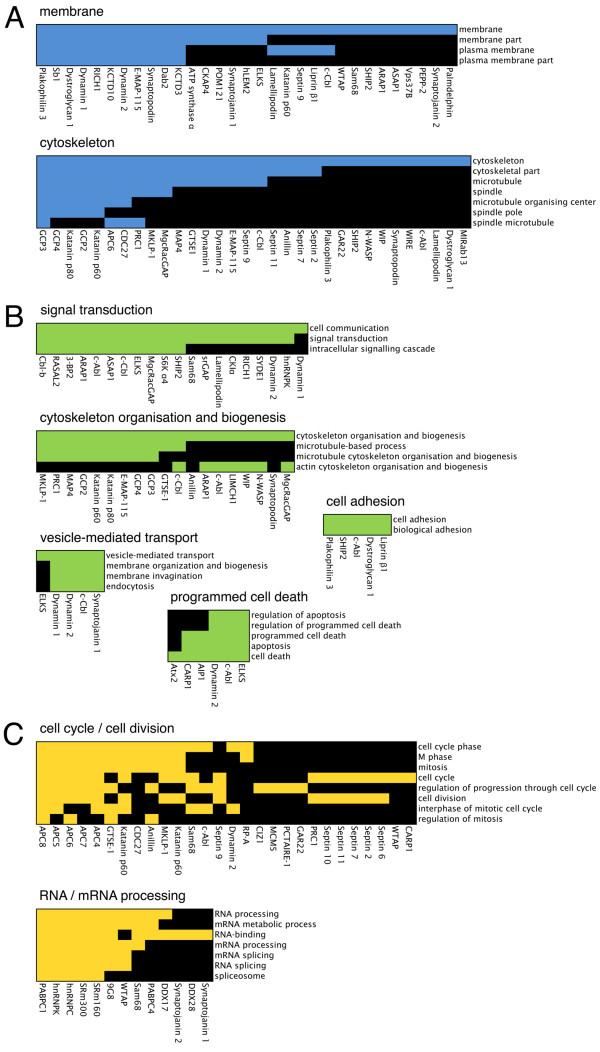
**Functional clusters of the proteins recruited by the SH3 domains of Ruk/CIN85 identified in DAVID 2.0 software**. A. Proteins clustered by cellular locations. B, C. Proteins clustered by the involvement in cellular processes (see text for details). Colour fields denote that corresponding associations between the proteins (given below each cluster) and the functional annotations (given at the right side of each cluster) have been positively reported. Black fields denote that corresponding associations have not been reported so far.

**Figure 4:**
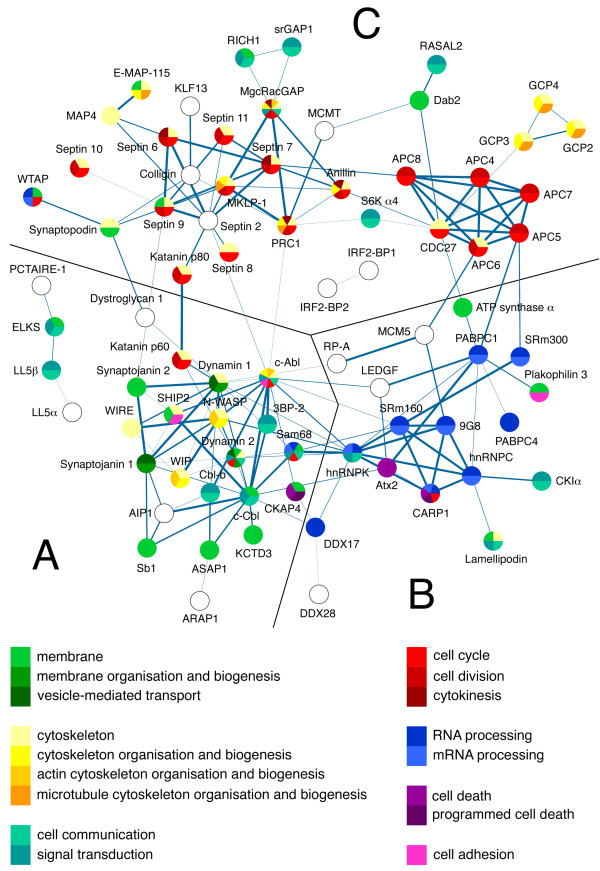
**Association network of the proteins recruited by the SH3 domains of Ruk/CIN85 obtained in STRING 8.0 software**. The functional annotations are mapped onto the network using different colours as indicated in the legend. A, B, C. The network clusters defined based on distinct functional profiles (see text for details). Blank circles indicate lack of annotation.

To obtain the functional profile of the proteins recruited by the SH3 domains of Ruk/CIN85, we clustered them according to functional annotations using DAVID (a database for annotation, visualisation and integrated discovery) [17]. In a complementary approach, we created the network of physical interactions and functional associations between these proteins using STRING (the meta-database search tool for the retrieval of interacting genes/proteins) [18] and mapped the resulting list of functional annotations onto this network. Both DAVID and STRING had their own limitations, and combination of these tools allowed to improve the quality of functional profiling. While DAVID analysis alone was more suitable for the annotation of functions enriched in the set of identified proteins in general (Fig. 3), results of STRING analysis showed interactions between the proteins and, in combination with the functional annotation, reflected potential multimeric protein complexes existing within the network (Fig. 4). Analysis performed by each of these tools covered approximately 75% of the proteins from the list of the Ruk/CIN85 interaction candidates, which is reasonably good result, because only about 20% of the human genes have been functionally annotated to date [19].

Initially, DAVID tool was used to cluster the proteins by their occurrence in different cellular locations. Two large meaningful clusters identified this way included proteins associated with the cytoskeleton and membranes (Fig. 3A). Next, the analysis of clustering of proteins by their involvement in different cellular processes was performed. The list obtained as the result of this analysis comprised of many functional clusters. As we expected, some of these clusters converged proteins with the cellular functions, that were previously associated with Ruk/CIN85, such as signal transduction, cytoskeleton organisation and biogenesis, vesicle-mediated transport, cell adhesion and programmed cell death (Fig, 3B). Some of these clusters apparently lacked some of the proteins, primarily because of their incomplete annotation in Gene Ontology database. This was particularly evident in the case of proteins taking part in vesicle-mediated transport. For example, the cluster of these proteins included c-Cbl and synaptojanin 1, but neither Cbl-b and synaptojanin 2 – their closely related homologues with similar functions, nor Dab2 and ASAP1 – the known Ruk/CIN85 interaction partners with well documented roles in membrane vesicle trafficking. At the same time, all these proteins were highly associated and located at the same cluster of protein association network obtained in STRING (Fig. 4A). In addition to proteins involved in vesicle-mediated transport, this cluster combined proteins involved in signal transduction and in organisation of actin cytoskeleton. Not surprisingly this cluster was also rich in the known Ruk/CIN85 interaction partners present in the network.

Unexpectedly, except for the protein clusters with Ruk/CIN85-related functions, both the resulting DAVID annotation list and the protein association network obtained in STRING contained clusters of proteins with the functions, which were not associated with this adaptor before. Two of these clusters combined proteins related to regulation of cell cycle and cell division (cytokinesis), and involved in RNA/mRNA processing (Fig. 3C; Fig. 4B, C), suggestive of previously unknown cellular roles of Ruk/CIN85.

## Discussion

### Ruk/CIN85 at the interface of membranes and the cytoskeleton

Growing body of evidence indicates that Ruk/CIN85 acts at the interface of membranes and the cytoskeleton. The synchronous rearrangements of membranes and associated cytoskeletal structures underlie many important cellular events such as cell motility and vesicle-mediated transport (membrane trafficking). Motile cells are constantly remodelling their membranes and the cytoskeleton to generate adhesive and protrusive structures required for migration, and cancer cells use the subversions of such structures to invade surrounding tissues. Coordinated remodelling of membranes and the cytoskeleton is also required to generate membrane vesicles and transport them between different intracellular membrane trafficking compartments.

Therefore, it was not surprising that Ruk/CIN85 had been previously found to be implicated in the regulation of both these processes, and majority of proteins recruited by the SH3 domains of Ruk/CIN85 identified here localise to membranes or cytoskeletal structures.

Recent studies suggest that physiologically diverse cellular processes, which require integration of membranes with the adjacent cytoskeleton, including vesicle-mediated transport, cell adhesion, migration and invasion may use compatible molecular mechanisms utilising similar sets of proteins and interactions. For this reason interactions between Ruk/CIN85 and such promiscuous proteins are of particular interest. For instance, previously Ruk/CIN85 has been implicated in the interaction with Arf GTPase-activating protein ASAP1/AMAP1, which is one of the core components of invadopodia in invasive cancer cells, and also regulates focal adhesions, circular dorsal ruffles and membrane trafficking [20,21]. Several such proteins are among the novel Ruk/CIN85 interaction candidates identified here. Two of them, N-WASP, a regulator of Arp2/3-mediated actin nucleation and elongation at membranes, and Dynamin 2, a mechanoenzyme capable to promote tubulation and fission of membranes, are worth of special attention. Both these proteins act in a coordinated manner at the protrusive structures of motile cells and at diverse membrane trafficking compartments [22-24]. Both are also the core components of invadopodia [25].

The results obtained so far indicate that Ruk/CIN85 acts at different cellular locations. However, the exact map is only beginning to emerge and analysis of effector molecules clustered by this adaptor may help to establish a better image. Variety of GTPase-activating proteins (GAPs) recruited by the SH3 domains of Ruk/CIN85 may serve as an excellent example. The GAPs for Rho and Arf family GTPases (RhoGAPs and ArfGAPs) are the substantial portion of proteins recruited by the SH3 domains of Ruk/CIN85 involved in membrane and cytoskeleton remodelling. Previously Ruk/CIN85 has been implicated in the interactions with two RhoGAPs (CAMGAP1, RICH1), one Arf GAP (ASAP1/AMAP1), and a Arf/Rho dual-specificity GAP (ARAP3) [10,26,27]. Another Arf/Rho dual-specificity GAP ARAP1, and several RhoGAPs (MgcRacGAP, srGAP, SYDE1) are among the Ruk/CIN85 interaction candidates identified here. ArfGAPs and RhoGAPs control activities of Arf and Rho family GTPases, which are the key regulators of membrane and actin organisation [20,28]. Noteworthy, the GAPs of both these classes outnumber the corresponding target GTPases, and are consequently regarded as their location-specific regulators [28].

Even though the majority of RhoGAPs recruited by the SH3 domains of Ruk/CIN85 are active towards Cdc42 GTPase, their sites of action are strikingly different. Scene for dual-specificity Arf/RhoGAP ARAP1 and ArfGAP ASAP1/AMAP1 is even more complex, because both act simultaneously at the cell interior and at the cell periphery. Taken together, the activities of the GTPase-activating proteins recruited by the SH3 domains of Ruk/CIN85 are distributed between a vast range of membranes and membrane compartments, ranging from the plasma membrane and protrusive/adhesive actin-rich structures such as focal adhesions, circular dorsal ruffles, lamellipodia, filopodia and invadopodia at the cell periphery to internal membrane trafficking compartments including the Golgi complex [20,21,26-33]. Diversity of the partner GAPs and the overlap of their localisation with cellular distribution of Ruk/CIN85 may therefore provide a plausible model explaining promiscuity of this adaptor towards different membranes and membrane trafficking compartments. According to this model, Ruk/CIN85 might act at the Golgi complex with ARAP1, whereas at different peripheral protrusive structures including lamellipodia and invadopodia with ASAP1/AMAP1, ARAP3 and RICH1. Ruk/CIN85 might also act with ARAP1 and ASAP1/AMAP1 along the endocytosis-recycling pathway. However, significance of Ruk/CIN85 for this process is in question and remains to be clarified (discussed in the next section).

### Ruk/CIN85 and vesicle-mediated trafficking

Our present data indicate that via the SH3 domains Ruk/CIN85 may recruit a number of protein molecules required for the proper formation and function of coated vesicles. Majority of these proteins, including dynamins, inositol-5'-phosphatase synaptojanin 1 and N-WASP are recruited to coated pits at the late steps of coated vesicle formation to complete vesicle fission [34-36].

In line with these observations, early studies implicated Ruk/CIN85 in the formation of clathrin-coated vesicles during the endocytosis of receptor tyrosine kinases, and particularly of the EGF receptors [4]. Proposed model assumed that Ruk/CIN85 clusters activated receptor complexes with A-type endophilines A1, A2 or A3 (endocytic machinery proteins, which use their BAR domains to sense and shape membranes) to facilitate membrane invagination during the formation of clathrin-coated vesicles. However, the conflicting results of two subsequent studies [12,13], and a lack of endogenous Ruk/CIN85 in both early and late endocytic compartments [11] questioned this view.

In our previous study, we observed only the weak presence of Ruk/CIN85 at compartments involved in clathrin-mediated endocytosis and sorting, while it was abundant at membrane trafficking compartments free of clathrin, primarily at the COPI-coated membranes and vesicles of the Golgi complex, and on some vesicular structures throughout the cell [11]. Based on these observations, it is reasonable to conclude that Ruk/CIN85 is likely to play more important role in the biogenesis of vesicles covered with coats other then clathrin, for instance of COPI-coated vesicles. Consistent with this, c-Cbl, the E3 ubiquitin ligase thought to engage Ruk/CIN85 to the activated EGF receptors [4], had been also implicated in processes at the Golgi complex [37], and thus could mediate the recruitment of Ruk/CIN85 to the Golgi membranes. Similarly, N-WASP in addition to its role in endocytosis, had been implicated in the regulation of actin polymerisation on COPI-coated membranes of the Golgi complex, and the mechanisms of N-WASP-mediated actin assembly observed at different coats were strikingly similar [38,39]. At the same time, consideration that Ruk/CIN85 acts exclusively at COPI-coated membranes have also some drawbacks. For instance, some of the Ruk/CIN85 interaction partners and candidates involved in endocytosis, such as A-type endophilins and dynamins, clearly do not function in biogenesis of COPI-coated vesicles [40]. Therefore, more a feasible explanation is that distinct pools of Ruk/CIN85 act at different membrane trafficking compartments through engagement of exclusive sets of proteins. This notion presumes, however, the existence of mechanism(s) to generate the populations of Ruk/CIN85 molecules with distinct properties. It is of interest, that our present data demonstrate that the SH3 domains of Ruk/CIN85 could bind cytoplasmic tyrosine kinase c-Abl, which had been implicated in the negative regulation of EGFR endocytosis via the impairment of c-Cbl-receptor interaction [41]. We therefore speculate that the recruitment of c-Abl by Ruk/CIN85 may serve as a mechanism to exclude some Ruk/CIN85 partners from the protein complexes, retain it (and c-Cbl) away from the plasma membrane and release for other cellular processes.

It has been previously suggested that scaffolding functions of Ruk/CIN85 in membrane transport are not limited to the formation of coated vesicles, but may also extend to the regulation of subsequent vesicle trafficking events [10]. In favour of this hypothesis, the SH3 domains of Ruk/CIN85 recruit diverse proteins implicated in distinct membrane trafficking events. Some of them, such as adaptor proteins AIP1/Alix and Dab2 are the known Ruk/CIN85 interaction partners [42,43]. Other proteins, including adaptor Vps37b, serine/threonine kinase PCTAIRE-1 or inositol-5'-phosphatase SHIP2 are the novel interaction candidates [44-46].

Intriguingly, Ruk/CIN85 itself displays several features characteristic for the components of vesicle coats. First, recruitment of Ruk/CIN85 to (the Golgi) membranes is modulated by Arf1 GTPase activity [11]. Second, Ruk/CIN85 can recruit variety of GTPase-activating proteins [[10,26,27] and this study]. Third, Ruk/CIN85 is able to oligomerise via its coiled-coil regions [47] and intermolecular SH3 domain – proline-rich region interactions [5]. Such homotypic oligomerisation may serve as an intrinsic mechanism to retain Ruk/CIN85 at membranes, making it similar to the coat constituent proteins. Thereby, we suggest that during different membrane trafficking events Ruk/CIN85, at least in part, may function as the component of vesicle coats. Since main coat component proteins are rarely dedicated to one particular function, this hypothesis may explain abundance and functional diversity of the proteins implicated in the interactions with Ruk/CIN85, as well as its presence at different membrane trafficking compartments and at distant vesicle transport intermediates scattered throughout the cell. This hypothesis is also in agreement with the previously proposed idea that Ruk/CIN85 acts at the consecutive steps of membrane trafficking events by continuous exchange of interaction partners [10].

### Ruk/CIN85 and cancer cell invasiveness

Current research indicates that Ruk/CIN85 may be an important regulator of cancer cell invasiveness [9]. It has been found that together with ASAP1/AMAP1 Ruk/CIN85 is necessary for the biogenesis of invadopodia [9]. These protrusive actin-rich membrane structures are found only in invasive cancer cells, but molecularly and functionally are similar to podosomes – the foot processes of several types of cells [25,48]. Proteolytic activities of invadopodia allow aggressive cancer cells to degrade extracellular matrix (ECM) and spread through surrounding tissues [49]. The interaction between Ruk/CIN85 and ASAP1/AMAP1 is critical to maintain the invasive phenotype of some breast cancer cell lines [9]. It was suggested that Ruk/CIN85 might regulate invadopodia via the recruitment of c-Cbl to ASAP1/AMAP1 and facilitation of the c-Cbl-mediated ubiquitination of this GTPase-acivating protein [9]. However, such regulatory role of Ruk/CIN85 at invadopodia may also span onto other targets. For instance, except ASAP1/AMAP1 Ruk/CIN85 interacts with inositol-5'-phosphatases synaptojanin 2 and SHIP1, which were also implicated in biogenesis of invadopodia and podosomes [50,51], and with non-receptor tyrosine kinase Src, which is the principal regulator of both cellular structures [49].

Our present data show that current view on the functional involvement of Ruk/CIN85 in invadopodia biogenesis may be oversimplified. Several proteins identified here as the novel interaction candidates for the SH3 domains of Ruk/CIN85 have well documented roles in this process. From these, N-WASP, N-WASP-interacting adaptor molecule WIP, and dynamin 2 are among the key regulators of invadopodia biogenesis [25]. N-WASP and WIP regulate invadopodia via the promotion of the Arp2/3-mediated assembly of branched actin arrays [49,52]. Despite of the proved importance of dynamin 2 to invadopodia, its functions there have not been yet defined. Two possibilities include regulation of associated membrane trafficking and/or regulation of actin assembly [25]. Additionally, most of the GTPase-activating proteins recruited by the SH3 domains of Ruk/CIN85 regulate Cdc42, which activates Arp2/3-mediated actin assembly via N-WASP, and have been recently shown to act upstream invadopodia formation [48].

Even though molecular mechanisms underlying invadopodia biogenesis remain mostly obscure, the short list of proteins identified so far indicates that invadopodia are closely related to podosomes and contain molecular components of focal adhesions [25]. Consistent with these relations, several of the novel Ruk/CIN85 interaction candidates identified here, such as non-receptor tyrosine kinase c-Abl, WIP-related N-WASP-binding protein WIRE, synaptopodin and cell adhesion receptor protein dystroglycan 1, were previously implicated in the regulation of these cellular structures [49,53,54]. Non-receptor tyrosine kinase c-Abl is the key regulator of actin at many protrusive and adhesive cellular structures including focal adhesions [55]. Presence of c-Abl at podosomes or invadopodia had not been studied yet. However, an indirect evidence connects its oncogenic BCR-fused variant (BCR/Abl) to the promotion of podosome formation in leukaemic cancer cells [56]. Recently mutant Abl kinase variants have been associated with the promotion of strongly invasive phenotype also in aggressive breast cancer cells [57]. Synaptopodin, which interacts also with CD2AP, and localises to focal adhesions and foot processes of podocytes, had been implicated in cell migration [58]. Transmembrane adhesion receptor Dystroglycan 1 had been recently found to modulate podosome formation in myoblasts [53]. Prominently, dystroglycan 1 regulates these cellular structures in complex with Tks5/FISH, the specific component of invadopodia and podosomes [49,59]. Although Tks5/FISH was not identified among Ruk/CIN85 interaction candidates, in our samples we found Tks4, recently discovered adaptor protein closely related to Tks5/FISH [60].

Of particular interest, Tks4 and Tks5 are the only specific regulators of invadopodia and podosomes identified so far [59,60]. Recent study by Oikawa et al. [61] showed that podosomes originate from focal adhesions via activation of Src kinase followed by accumulation of PI(3,4)P2, targeting of Tks5 to podosome precursors and augmentation of Arp2/3-mediated actin assembly by N-WASP molecules recruited to Tks5 platform. Although this model is too simplistic [60], it describes the sequential steps required for the biogenesis of podosomes (and probably also of invadopodia), including activation of Src, production of PI(3,4)P2 and promotion of Arp2/3-mediated actin assembly by N-WASP. The most likely pathway of PI(3,4)P2 production is through dephosphorylation of PI(3,4,5)P3 produced sequentially by PI 3-kinase and inositol-5'-phosphatases, including synaptojanins and SHIP2 [61]. In addition, similarly to Tks5/FISH, Tks4 binds predominantly to PI(3)P and PI(3,4)P2 [60]. Strikingly, through the different protein interaction modules Ruk/CIN85 appears to be able to cluster all of these molecules, including PI 3-kinase [1], synaptojanins, SHIP2, Tks4, and also N-WASP. Therefore, Tks4 and Ruk/CIN85 together are probably able to assemble a multimeric protein complex harbouring activities required to produce PI(3,4)P2 (and hence stabilise Tks4 recruitment to membranes), as well as to regulate Cdc42 and promote Arp2/3-mediated actin assembly. Such complex therefore would fulfil all requirements for the successful initiation of podosome formation in response to Src kinase activity.

Hence, although further experiments are obviously necessary, the existing data suggest that Ruk/CIN85 may control biogenesis of podosomes and invadopodia directly via the augmentation of actin assembly. Noteworthy, although it is not the general case for all cell models, the strong association between Ruk/CIN85 and actin-rich structures including focal adhesions had been found in astrocytes [6]. Also, a recent study revealed the actin-bundling properties of this adaptor [47]. At the same time, Ruk/CIN85 might participate in vesicle-mediated transport associated with these cellular structures. This second non-exclusive possibility is supported by the documented roles of Ruk/CIN85 in membrane trafficking. In this respect the involvement of Ruk/CIN85 in the Golgi-associated membrane trafficking is worth of particular attention because of the spatial relationship between Golgi complex and invadopodia [48,49]. The notion that vesicle-mediated transport from the Golgi area to invadopodia might be important for the delivery of proteases and other structural components to invadopodia is supported by the observed polarisation of the Golgi complex towards invadopodia, and the dependence of their ECM-degrading activities on the Golgi integrity [48,49].

If Ruk/CIN85 can indeed function both in membrane trafficking and actin remodelling in the bifacial manner, then the shifts of its attraction towards one of these processes in different cell types should be governed by certain regulatory mechanisms. Due to the fact that the appearance of malignant and invasive cell phenotypes (including ability to generate invadopodia) often relies on *de novo *re-wired signalling pathways [62], search for the pathologic subversions of such regulatory processes in invasive cancer cells may contribute to better understanding of how exactly Ruk/CIN85 is redirected to function in invadopodia biogenesis.

### Ruk/CIN85 and other cellular processes

Except for the proteins participating in membrane and actin remodelling, among the proteins recruited by the SH3 domains of Ruk/CIN85 we distinguished two groups of proteins with cellular functions, which had not been associated with Ruk/CIN85 before. These included proteins related to cell cycle and cell division, as well as proteins involved in RNA/mRNA processing. Additionally, the functional cluster associated with the cytoskeleton organisation and biogenesis contained many proteins with microtubule-related functions.

Although the participation of Ruk/CIN85 in remodelling of actin cytoskeleton had been demonstrated in several studies, current information on its potential role in organisation of microtubules is very limited. Therefore, it is of interest that the SH3 domains of Ruk/CIN85 recruit several proteins involved in the regulation of microtubule dynamics. This primarily applies to GCP2, GCP3 and GCP4 proteins which are the main components of large and small γ-tubulin complexes (γTuSC and γTuRC) responsible for the nucleation of microtubules at centrosomes and at other cellular structures [63]. Several lines of evidence indicate that γ-tubulin complexes may participate in the non-centrosomal nucleation of microtubules on membranous cellular compartments, including the Golgi complex, and that it is required for the maintenance of the Golgi structure [63]. Three other interaction candidates, LL5β, ELKS and CKAP4, have been implicated in the attachment of microtubules to membranes [64,65]. Noteworthy, LL5β and ELKS act together to link microtubules with the cell cortex, focal adhesions and the plasma membrane at the leading edge of motile cells [64]. Although our samples contained also several microtubule-binding proteins with undefined functions such as DDA3, E-MAP-115 and Gar22 [66-68], in general it appears that many of the Ruk/CIN85 interaction candidates are implicated in the regulation of microtubule dynamics act at membranes. Thereby, the most straightforward explanation for our findings is that via the recruitment of such proteins Ruk/CIN85 may participate in the regulation of microtubule dynamics at membrane compartments.

Similarly, in our study the SH3 domains of Ruk/CIN85 recruited the set of proteins involved in processing of RNA/mRNA molecules. Majority of these proteins including SRm160, SRm300, Sam68, poly(A)-binding proteins PABP1 and PABP4, and heterogeneous nuclear ribonucleoproteins hnRNPK and hnRNPC are involved in different aspects of pre-mRNA splicing [69-72]. Importantly, similarly to Ruk/CIN85, some of these proteins had been functionally linked to the regulation of actin dynamics and cell migration. For instance, hnRNPK had been implicated in the direct interaction with and the regulation of N-WASP at the cell periphery [73]. Of even more interest, three of these proteins including hnRNPK, Sam68 and PABP1 are present at focal adhesions, and PABP1 was found to regulate cell spreading and migration through the export of specific pre-mRNA molecules to these cellular structures [74,75].

Finally, many of the proteins recruited by the SH3 domains of Ruk/CIN85 identified here are known to be involved in the regulation of cell cycle, and particularly of cell division (cytokinesis). Four of these proteins, including anillin, MgcRacGAP, MKLP-1 and PRC1, constitute the core machinery controlling formation and maintenance of the central spindle and the actomyosin contractile ring during cytokinesis [76-78]. In addition, the set of identified cell division-associated proteins included both subunits of microtubule-severing heterodimeric ATPase katanin, several subunits of E3 ubiquitin ligase Anaphase Promoting Complex (APC/Cylosome), microtubule-associated protein MAP4 and septins – GTP-binding proteins able to assemble into highly dynamic filamentous structures associated with membranes, microtubules and actin filaments [79]. While microtubule-severing activity of katanin is required to control spindle length [80], APC/Cyclosome is one of the principal regulators of cell cycle progression responsible predominantly for final steps of mitosis and cytokinesis [81]. Anillin, MgcRacGAP and PRC1 are all known substrates for APC/Cyclosome [81,82]. In turn, septins are thought to co-operate with MAP4 and Anillin in the regulation membrane organisation and vesicle trafficking at the cleavage furrow necessary for cytokinesis completion [83].

Although the function(s) of Ruk/CIN85 in cytokinesis is (are) hard to envisage, its participation in this process is feasible for several reasons. First, CD2AP/CMS, the close homolog of Ruk/CIN85 (and its interaction partner), is known to bind anillin and have been already implicated in the regulation of cytokinesis [84]. Second, membrane trafficking processes required for the addition of membranes to the cleavage furrow and to the midbody, as well as vital for the completion of cell division utilise many proteins normally involved in vesicle-mediated transport of interphase cells [85]. These proteins include not only dynamins but also another known Ruk/CIN85 interaction partner AIP1/Alix [86,87]. And third, a recent study have demonstrated the crucial role of the Golgi-associated membrane trafficking and of the Golgi-derived vesicles in this process [88].

## Conclusion

Results of our study show complexity of protein interaction networks organised on the SH3 domains of Ruk/CIN85 by identification of a number of proteins recruited by the SH3 domains this adaptor. Many of them are known to be involved in the regulation of membranes and cytoskeletal structures required for vesicle-mediated transport and cancer cell invasiveness. Our data support the notion that Ruk/CIN85 regulates vesicle-mediated transport and cancer cell invasiveness through the assembly of multimeric protein complexes governing coordinated remodelling of membranes and underlying cytoskeletal structures, and imply important roles of this adaptor in formation of coated vesicles and biogenesis of invadopodia. Finally, our study points to potential involvement of potential Ruk/CIN85 in other cellular processes, in particular in cell division, and may indicate directions for future research.

## Methods

### Plasmids and reagents

Plasmids for expression of the glutathione S-transferase (GST)-fusion SH3 domains of Ruk/CIN85 in *E. coli *were provided by Dr. Vladimir Buchman (Cardiff School of Biosciences, Cardiff, United Kingdom; see ref. [5] for details).

Cell culture reagents were from Invitrogen (Carlsbad, CA). Glutathione Sepharose 4B was from Amersham Biosciences AB (Uppsala, Sweden). 10% Tris-HCl Ready Gel precast gels were from Bio-Rad Laboratories (Hercules, CA). Sequencing Grade Modified Trypsin was from Promega (Madison, WI). 0.1% formic acid (FA) solutions in water and acetonitrile (ACN) were acquired from Mallinckrodt Baker (Phillipsburg, NJ). All other reagents were from Sigma-Aldrich (St. Louis, MO).

### Cell culture

Human cervix adenocarcinoma HeLa cells obtained from European Collection of Cell Cultures were grown in plastic plates at 37°C and 5% CO_2 _in DMEM medium supplemented with GlutaMAX-I, 10% foetal bovine serum (FBS), 50 U/ml penicillin and 50 μg/ml streptomycin antibiotics.

### Purification of GST-fusion proteins

E. coli BL21(DE3)pLysS bacteria (Novagen, EMD Biosciences, Madison, WI) transformed with the plasmids for expression of the GST-fused SH3 domains of Ruk/CIN85 or of GST alone were grown in LB medium with 1% glucose until OD_600 _= 0.6 and then expression of the proteins was induced by 0.2 mM IPTG. After incubation for additional 2 hours at 25°C the bacterial cultures were harvested by centrifugation and purification of expressed proteins was performed on Glutathione Sepharose 4B according to the manufacturer's instruction (Amersham Biosciences AB, Uppsala, Sweden).

### GST pull-down experiments

HeLa cells pre-washed twice with ice-cold PBS were harvested in the buffer of 20 mM Tris (pH 7.5), 150 mM NaCl, 5 mM EDTA, 5% glycerol, 0.5% Triton X-100 supplemented with Complete protease inhibitor cocktail (Roche Diagnostics GmbH, Mannheim, Germany) and Phosphatase inhibitor cocktails 1 and 2 (Sigma, St. Louis, MO) and lysed for 30 min on ice. The lysates were clarified by centrifugation at 18000 g for 20 min at 4°C. To remove proteins non-specifically binding to Glutathione Sepharose 4B or to glutathione S-transferase, collected supernatant was pre-cleared by incubation with GST alone bound to Glutathione Sepharose 4B beads for 2 hours at 4°C, and the beads were removed by centrifugation at 18000 g for 10 min at 4°C. Equal amounts of the GST-fusion SH3 domains of Ruk/CIN85 or GST alone bound to Glutathione Sepharose 4B (approximately 10 μg of protein to 40 μl of beads per sample) equilibrated with the same buffer were incubated for 4 hours at 4°C with equal amounts of the pre-cleared lysate of HeLa cells. The beads were washed five times in the buffer containing 20 mM Tris (pH 7.5), 150 mM NaCl, 5 mM EDTA, 5% glycerol, and 0.1% Triton X-100 and then boiled in SDS-loading buffer.

### Sample preparation and protein identification by LC-MS/MS

The samples obtained in the pull-down experiments were separated on SDS-PAGE in 10% Tris-HCl precast gels and visualised by Coomassie R-250 staining. After the electrophoretic separation of samples, equal pieces were cut from experiment (GST-fusion SH3 domains) and control (GST alone) gel lanes as indicated in Additional file 1.

Prior to the analysis excised gel slices were subjected to the standard procedure of in-gel trypsin digestion, during which proteins were reduced with 100 mM DTT for 30 min at 56°C, alkylated with iodoacetamide in darkness for 45 min at room temperature, and digested overnight with 10 ng/ul trypsin. Peptides were eluted from gel with the water solution of 0.1% FA and 2% ACN. The resulting peptide mixtures were applied to RP-18 pre-column (Waters, Milford, MA) using water containing 0.1% FA as a mobile phase and then transferred to a nano-HPLC RP-18 column (internal diameter 75 μM, Waters, Milford MA) using ACN gradient (0 – 30% ACN in 45 min) in the presence of 0.1% FA at a flow rate of 250 nl/min. The column outlet was coupled directly to the ion source of LTQ FTICR mass spectrometer (Thermo Electron Corp., San Jose, CA) working in the regime of data-dependent MS to MS/MS switch. A blank run ensuring absence of cross-contamination from previous samples preceded each analysis.

### Analysis of mass spectrometry data

After pre-processing the raw data with Mascot Distiller software (version 2.1.1, Matrix Science, London, UK), obtained peak lists were used to search the non-redundant protein database of the National Centre for Biotechnology Information (NCBI) version 20070428 (4874565 sequences, 192489 human sequences) using the Mascot search engine (version 2.2, 8-processors onsite license) (Matrix Science, London, UK) with the following search parameters: taxonomy restriction – *Homo sapiens *(human), enzyme specificity – semi-trypsin, permitted number of missed cleavages – 1, fixed modification – carbamidomethylation (C), variable modifications – carbamidomethylation (K) and oxidation (M), protein mass – unrestricted, peptide mass tolerance – ± 40 ppm, fragment mass tolerance – ± 0.8 Da, max missed cleavages – 1. The searches were restricted to *Homo sapiens *sequences because HeLa cells used in our experiments originated from the human. Only proteins for which at least two peptides with Mascot cut-off scores ≥ 48, indicating identity or extensive homology of peptide (p ≤ 0.05), were considered as the positive identifications.

The resulting protein lists for the experiment (GST-fusion SH3 domains) samples were compared with the control (GST alone) lists to exclude proteins non-specifically binding to GST or Glutathione Sepharose 4B. Protein hits scoring as the positive identifications in the controls were in all cases removed from the lists, even if the corresponding hits from the experiment samples matched larger number of peptides. For the protein hits matching two or more database entries (e.g. several protein isoforms or products of a same gene carrying single amino acid substitutions) accession number of the best annotated entry were listed in Table 1, all other accession numbers were reported in Additional file 2. Partial protein sequences without N-terminal methionine, derived e.g. from the conceptual translation of partial mRNA sequences were in all cases excluded from the analysis. Additional file 2 includes also long and alternative names, as well as accession numbers of proteins in UniProt Knowledgebase Release 14.0 (22 July 2008).

### Analysis of protein modular architectures

Analysis of modular architectures of the identified proteins was performed by querying their sequences against InterPro version 19.0 database using InterProScan software [89] and by searches in Ensembl release 52 database . The short names of the modules found in the identified proteins were listed in Table 1, for the long names refer to Additional file 3.

### Scansite analysis

Presence of potential recognition consensus sites for the SH3 domains of Ruk/CIN85 in protein sequences was analysed using Scansite version 2.0 software [90]. Scansite matrices for the SH3 domains of Ruk/CIN85 (see Additional file 5) were constructed in accordance with the guidelines provided by software developers. For the construction of each matrix, we used the experimental data on consensus peptide composition obtained previously by Kurakin et al. [2] through target-assisted iterative screening (TAIS) of bacteriophage-displayed random peptide libraries. In brief, the arginine residue (R) flanking peptide ligand core sequence recognised by all the SH3 domains of Ruk/CIN85 (PX(P/A)XXR) was selected as the invariant fixed position residue in the matrices. Matrix scores for different amino acids in each position within ligand binding core were calculated from their frequencies in the peptides selected by TAIS. Very low score values (0.1) were applied to allow for the presence of alternative amino acid residues in non-critical (second, fourth and fifth) positions within the ligand binding core. The analysis was performed on the protein sequences corresponding to the entries listed in the Table 1.

### Functional annotation clustering and association network analyses

Analysis of functional enrichment within the identified proteins was performed in DAVID 2.0 software [17] according to the standard protocol [91]. Functional annotation clustering tool was used to avoid the occurrence of highly related or redundant terms in the results. The combined list of official gene symbols corresponding to the identified proteins was used for input (see Additional file 6). Association network analysis was performed by STRING 8.0 software [18] with the following analysis parameters: species – *Homo sapiens*, confidence level – 0.200, active prediction methods – all, and using combined list of Ensembl gene IDs for input (see Additional file 6). The resulting protein association network was visualised in Cytoscape 2.6.1 software [92]. The functional annotations were mapped onto the network using the same software.

## Abbreviations

Ruk/CIN85: Regulator of ubiquitous kinase/c-Cbl-interacting protein of 85 kDa; CD2AP: CD2-associated protein/Cas ligand with multiple SH3 domains; LC-MS/MS: liquid chromatography-coupled tandem mass spectrometry; GST: glutathione S-transferase.

## Competing interests

The authors declare that they have no competing interests.

## Authors' contributions

SH conceived the study, participated in its design, participated in protein purification, GST pull-down experiments, as well as performed analysis of the LC-MS/MS data, functional annotation clustering and other bioinformatics analyses. YR participated in protein purification and GST pull-down experiments. AM performed LC-MS/MS. LD had been involved in drafting and critical revision of the manuscript. MJR participated in design of the study, coordinated it and helped to draft the manuscript. All of the authors read and approved the final manuscript.

## Supplementary Material

Additional file 1 **Modular organisation of Ruk/CIN85 and representative SDS-PAGE gel resulting from GST pull-down experiment**. A. Modular organisation of Ruk/CIN85 adaptor molecule. B. Representative SDS-PAGE gel loaded with 1/5 of each sample resulting from GST pull-down experiment. For the LC-MS/MS analysis proteins were separated on a parallel SDS-PAGE gel loaded with 4/5 of each sample. Blank tracks were left between the loaded lanes to avoid cross-contamination. Locations of the excised gel pieces are outlined with colour boxes. Three corresponding gel slices with the size ranges of 75 – 175 kDa, 45 – 75 kDa and 33 – 45 kDa were excised from each lane. Click here for file

Additional file 2 **Proteins recruited by the SH3 domains of Ruk/CIN85 identified by the LC-MS/MS**. The table lists the proteins recruited by the SH3 domains of Ruk/CIN85 identified by the LC-MS/MS. *GI *– protein genbank identifier; *Name *– short protein name; *Sample *– sample where protein was identified (refer to Additional file 1 for details); *Score *– Mascot protein score; *Mr *– protein molecular weight; *u.p*. – number of unique peptides matched to protein sequence; *% c*. – % of protein sequence covered by matched peptides; *Uniprot *– protein accession number in UniProt Knowledgebase. The long and/or alternative names, as well as all additional genbank identifiers for the identified proteins are listed in the two last columns. Click here for file

Additional file 3 **Long names of module/domain abbreviations mentioned in the Table **1. The table lists the long names of modules/domain abbreviations mentioned in the Table 1. Click here for file

Additional file 4 **Circular phylogram of all the SH3 domains found in mice and men.** The figure shows the circular phylogram of all the SH3 domains found in mice and men. The clade harbouring the SH3 domains of Ruk/CIN85 is indicated in green. Magnify. Click here for file

Additional file 5 **Scansite matrices used for prediction of potential recognition consensus sites for the SH3 domains of Ruk/CIN85 in the identified proteins.** The data provided represent the Scansite matrices used for prediction of potential recognition consensus sites for the SH3 domains of Ruk/CIN85 in the identified proteins. Click here for file

Additional file 6 **ENSEMBL IDs of the genes encoding the identified proteins**. The table lists ENSEMBL IDs of the genes encoding the identified proteins. Click here for file
