# Supplementary figures and images for: Proteins recruited by SH3 domains of Ruk/CIN85 adaptor identified by LC-MS/MS
Source: Proteome Sci. 2009 Jun 16;7:21. doi: 10.1186/1477-5956-7-21 (PMC2702278; doi:10.1186/1477-5956-7-21)

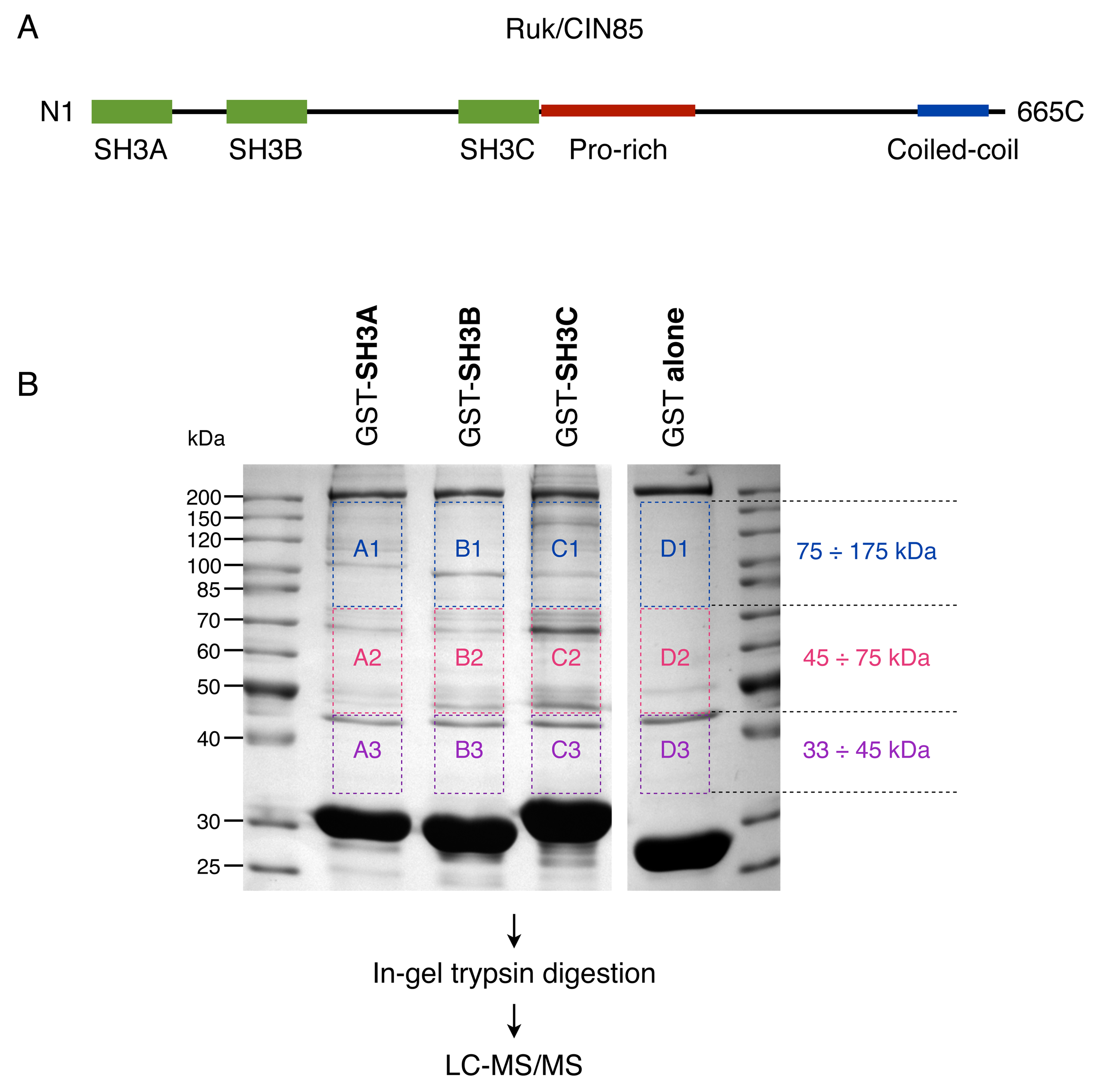

Supplement: Additional file 1 — Modular organisation of Ruk/CIN85 and representative SDS-PAGE gel resulting from GST pull-down experiment. A. Modular organisation of Ruk/CIN85 adaptor molecule. B. Representative SDS-PAGE gel loaded with 1/5 of each sample resulting from GST pull-down experiment. For the LC-MS/MS analysis proteins were separated on a parallel SDS-PAGE gel loaded with 4/5 of each sample. Blank tracks were left between the loaded lanes to avoid cross-contamination. Locations of the excised gel pieces are outlined with colour boxes. Three corresponding gel slices with the size ranges of 75 – 175 kDa, 45 – 75 kDa and 33 – 45 kDa were excised from each lane. [file 1477-5956-7-21-S1.png]

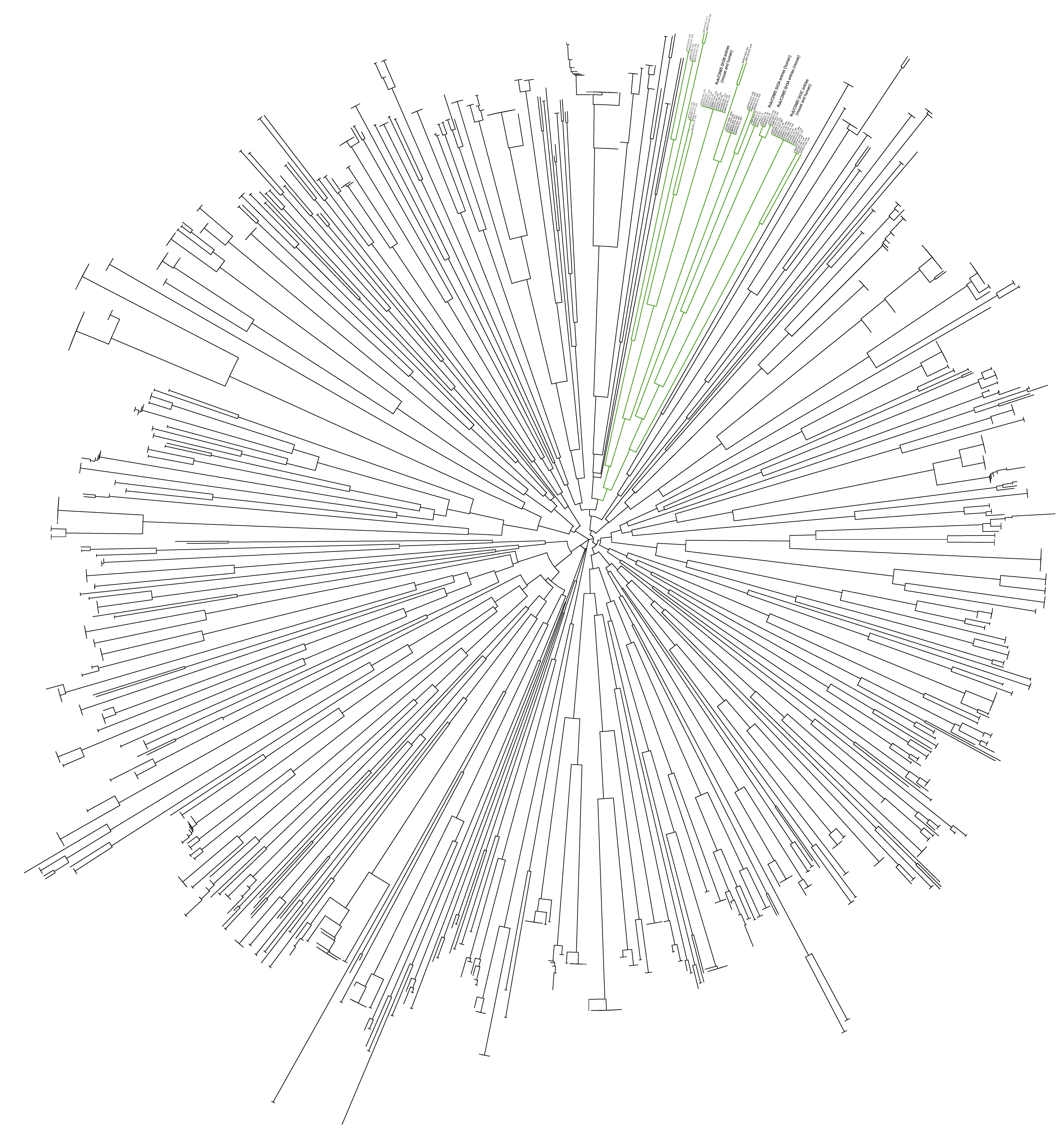

Supplement: Additional file 4 — Circular phylogram of all the SH3 domains found in mice and men. The figure shows the circular phylogram of all the SH3 domains found in mice and men. The clade harbouring the SH3 domains of Ruk/CIN85 is indicated in green. Magnify. [file 1477-5956-7-21-S4.png]
